# Paper Supercapacitor Developed Using a Manganese Dioxide/Carbon Black Composite and a Water Hyacinth Cellulose Nanofiber-Based Bilayer Separator

**DOI:** 10.1021/acsami.3c11005

**Published:** 2023-10-28

**Authors:** Mustehsan Beg, Keith M. Alcock, Achu Titus Mavelil, Dominic O’Rourke, Dongyang Sun, Keng Goh, Libu Manjakkal, Hongnian Yu

**Affiliations:** School of Computing and Engineering & the Built Environment, Edinburgh Napier University, Merchiston Campus, EH10 5DT Edinburgh, U.K.

**Keywords:** eco-friendly, cellulose, separator, supercapacitor, paper energy storage, flexible

## Abstract

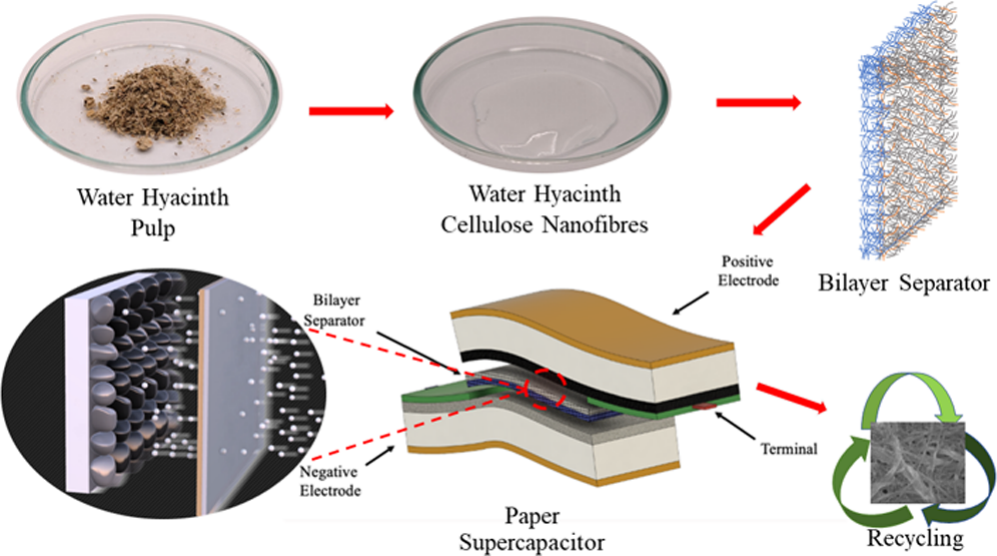

Flexible and green energy storage devices have a wide range of applications in prospective electronics and connected devices. In this study, a new eco-friendly bilayer separator and primary and secondary paper supercapacitors based on manganese dioxide (MnO_2_)/carbon black (CB) are developed. The bilayer separator is prepared via a two-step fabrication process involving freeze–thawing and nonsolvent-induced phase separation. The prepared bilayer separator exhibits superior porosity of 46%, wettability of 46.5°, and electrolyte uptake of 194% when compared with a Celgard 2320 trilayer separator (39%, 55.58°, and 110%). Moreover, lower bulk resistance yields a higher ionic conductivity of 0.52 mS cm^–1^ in comparison to 0.22 mS cm^–1^ for the Celgard separator. Furthermore, the bilayer separator exhibits improved mean efficiency of 0.44% and higher specific discharge capacitance of 13.53%. The anodic and cathodic electrodes are coated on a paper substrate using MnO_2_/CB and zinc metal-loaded CB composites. The paper supercapacitor demonstrates a high specific capacitance of 34.1 mF cm^–2^ and energy and power density of 1.70 μWh cm^–2^ and 204.8 μW cm^–2^ at 500 μA, respectively. In summary, the concept of an eco-friendly bilayer cellulose separator with paper-based supercapacitors offers an environmentally friendly alternative to traditional energy storage devices.

## 1 Introduction

Water hyacinth is typified as a herbaceous, free-floating, invasive aquatic plant that can be converted to high-value cellulose. Water hyacinth cellulose is characterized by its high cellulose content (40–60%) and is an inexhaustible, natural, renewable, and biodegradable source of raw material.^1^ Furthermore, cellulose is a linear, stereoregular, semicrystalline polysaccharide composed of β(1 → 4)-linked d-glucose units.^2,3^ The cellulose hierarchical structure leads to and is composed of nanofibers and their bundles, called microfibers. Moreover, the nanofiber’s high crystalline structure and aspect ratio exhibit impressive mechanical strength, barrier properties, and orientation ability.^2−4^ The unique property of cellulose leads to its application in the development of paper or fiber-based electronic devices including sensors,^5,6^ energy generators,^7,8^ and energy storage.^9−11^ Over the past decade, there has been an increasing interest in the development of paper-based flexible energy storage devices.^12−14^ The use of paper is motivated by its inherent features such as the mechanical strength, flexibility, porosity, large specific surface area, low cost, biocompatibility, and environmental friendliness. Furthermore, research has demonstrated that free-standing paper electrodes, with a thickness greater than 15 μm, can provide a substantial amount of energy and power density.^15^ Moreover, paper’s inherent rough and porous surface is advantageous for both electron manipulation and ion transportation throughout the structure, especially within the electrode, ultimately leading to high-power density.^16^ In a paper-based energy storage device, one of the main components is the separator; its main purpose is the safety of the device via separation of the electrodes while being permeable to the electrolyte. Furthermore, the compound annual growth rate of separators is projected to reach 16.1% by 2027 with petroleum-based polyethylene (PE) as the primary material used in electrochemical energy storage devices. Moreover, the cost of the separators can exceed 20% of the total device mainly due to its complex manufacturing processes and expensive raw materials.^17−19^

Cellulose-based separators have been previously investigated as substitute materials for environmentally harmful petroleum-based polymeric commercial separators. In a previous study on the processing and characterization of a water hyacinth cellulose nanofibers (WHCNF) separator via freeze–thawing, it was demonstrated that a high ratio of WHCNF (95 wt %) increased the porosity, electrolyte uptake, and wettability of the separator. This makes it a promising and sustainable option for a separator material.^20^ Furthermore, freeze-drying,^21−25^ nonsolvent-induced phase separation (NIPS),^24,26−28^ thermally induced phase separation,^29,30^ and electrospinning^31−33^ are commonly used processes to fabricate CNF separators. An ultrathin (19 μm) cellulose nanofiber (CNF) separator is fabricated to suppress the polysulfide shuttle effect and dendrite growth for the lithium–sulfur battery (LSB) using isopropanol/water suspension through a vacuum filtration process. The CNF polar oxygen functional group helps to immobilize the polysulfides and suppress the formation of dendrites on the lithium metal electrode, and isopropanol controls the highly porous structure (98.05%) of the membrane. The results show the CNF with IPA/water vol/vol % ratio of 95:5 discharge capacity increased by 1.4 and 1.3 times after 100 cycles when compared to the polypropylene (PP) separator.^34^ In another work, a three-dimensional (3D) interconnected network was used from a H-bond cross-linked cellulose/PI-COOH (1:10, vol %) composite by successive electrospinning for superior mechanical and thermal properties of the separator. The H-bond improves the affinity and wettability of the separator for the electrolyte. Cellulose/PI-COOH composite shows excellent cycle and rate performance in the lithium-ion cell.^35^ A biofiller biomembrane that is also suitable with a lithium-ion battery consists of 0.5, 1, and 2 wt % sulfonated cellulose blended with PLA/PBS composite using a spin-coating and phase inversion technique to improve thermal stability and electrochemical performance. The 2 wt % SC/biomembrane exhibits higher porosity (87%), electrolyte uptake (290.6%), and ionic conductivity of 3.24 mS cm^–1^ compared to the PP separator.^36^ In addition to this, a highly porous novel zeolitic imidazolate framework-8@bacterial cellulose/aramid nanofibers composite separator was used via the facile in situ synthesis and subsequent filtration process to achieve high safety and excellent electrochemical performance.^37^ A doctor blade fabricated separator is also reported^38^ in which a thermally stable and highly porous separator (83.6%) based on cellulose acetate utilized glycolic acid to create pores by applying water pressure for low-cost processing.

In addition, with a separator in a paper-based energy storage device, the active energy storage materials and their electrode fabrication are critical. There are various materials employed for paper-based supercapacitors (SC) fabrication: a few of them are listed in Table S1. SC are energy storage devices that can be categorized into two main types: electric double-layer capacitor (EDLC), which stores electrical energy by intercalating charges at the electrode–electrolyte interface, forming the double layer of charges, and pseudo-capacitors that use faradaic reactions to store electric energy.^39−43^ Paper-based SC offers a range of advantages, including flexibility, low cost, environmental sustainability, high surface area, scalability, rapid charge/discharge, and safety. The development of flexible-based energy storage devices has become a hot research topic due to the high demand for next-generation portable and wearable electronics, which will power future developments in the Internet of Things and modern energy storage technologies.^44−50^

**Figure 1:**
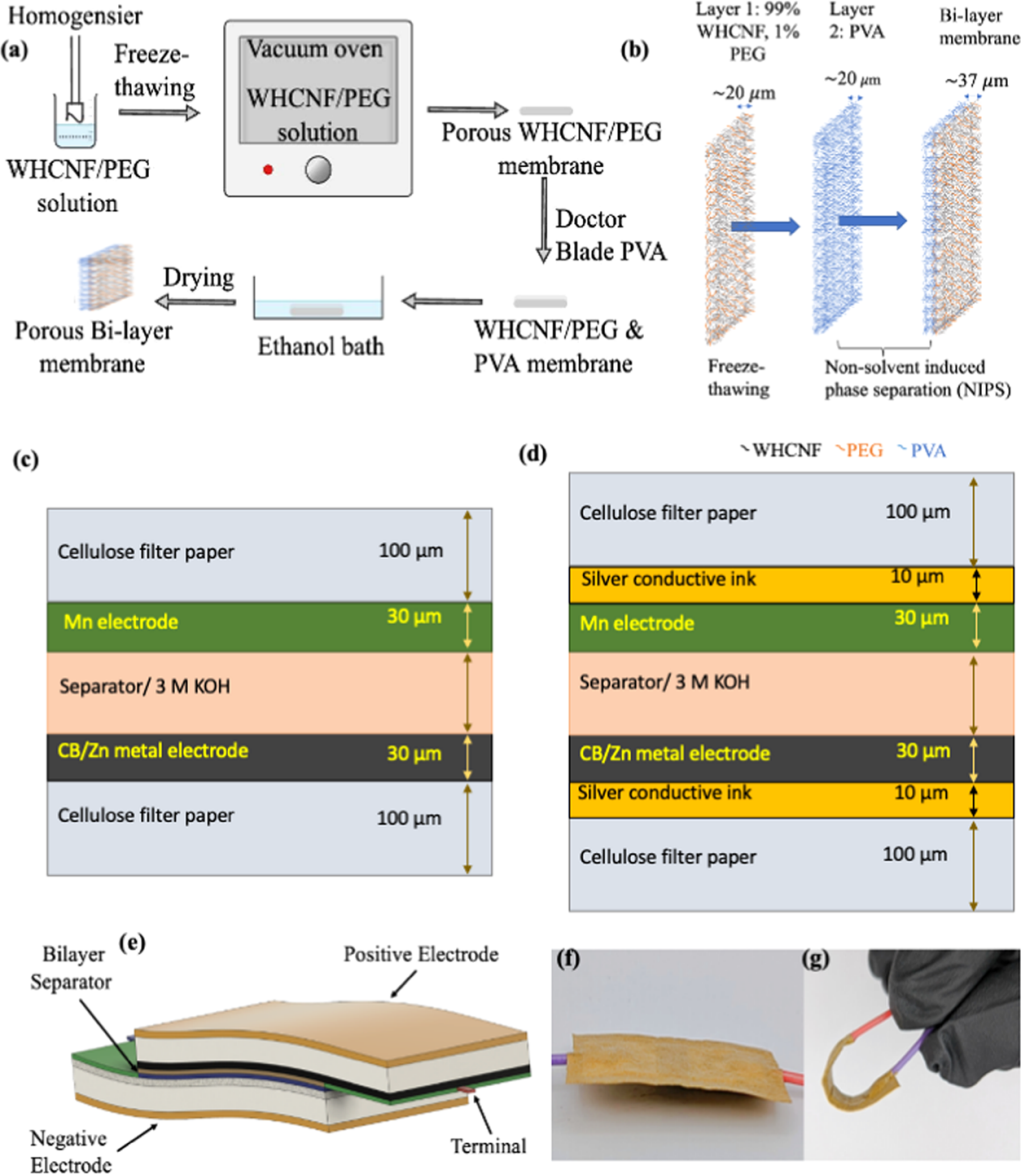
(a, b) Schematic illustration of the procedure of bilayer separator development and its structure; (c, d) 2D schematic illustration of the primary and secondary paper SCs; (e) schematic diagram of the flexible SC; and (f, g) showcase of the SC and its bendability.

In this work, we developed a new high-performance flexible energy storage device, specifically a SC. To achieve this, we designed a novel eco-friendly bilayer separator using WHCNF. Furthermore, we prepared new active electrodes consisting of an MnO_2_/carbon composite for the cathode and carbon black with Zn metal as an anode. For separator development, water hyacinth stems were collected from a freshwater lake in Taman Tasik Seri Aman, Puchong, Malaysia, and then cellulose was extracted from the stem and converted to cellulose nanofibers.^51^ The bilayer WHCNF separator was prepared by fabricating the base layer by a freeze–thawing process, comprising of WHCNF and poly(ethylene glycol) 4000 (PEG). PEG is a nontoxic, inexpensive, stable, easy-to-use, and biodegradable plasticizer that imparts cross-linking, flexibility, increased wettability, and electrolyte uptake to the separator.^20^ Layer two via doctor blade and NIPS is made of nontoxic, low environmental impact, and low-cost poly(vinyl alcohol) (PVA), which includes its high chemical resistance, aqueous solubility, and biodegradability.^52,53^ At high temperatures, the pure PVA layer melts and the pores close, stopping further ion transportation and the flow of current, also known as the separator shutdown effect for the safety of the device. A schematic presentation of the preparation of the bilayer separator and its layer structure is shown in Figure 1a,b. Furthermore, the WHCNF bilayer separator performance is compared with a commercially available Celgard 2320 trilayer separator consisting of three layers of polyolefin polymers (PP/PE/PP).

The paper SC is fabricated to evaluate the performance of the WHCNF bilayer separator, assessing its compatibility with the MnO_2_/carbon black composite-based electrode. Here, the carbon black with Zn (Zn/CB) metal increases its electrical conductivity as a negative electrode. These new composite paste-based electrodes are applied using a doctor blade onto the paper substrate. The information regarding the electrical conductivity of the electrode is discussed in the Supporting Information. In this study, we developed two types of SC using the above anodic and cathodic materials, and the device’s electrode layout is shown in Figure 1c,d, including its dimensions (material information provided in the Supporting Information). These SCs are termed primary SCs (single use, Figure 1c) and secondary SCs (which can be used for multiple charging/discharging cycles, as shown in Figure 1d), in which the active electrode is printed on top of the silver paste (current collector) to enhance performance. To assess the performance of a paper SC with the bilayer separator, SC is put through a long charge and discharge cycle to assess the Coulombic efficiency and discharge capacitance. Compared to previously reported paper-based SC, the newly developed SC designed in this study exhibits high energy density. Its properties are evaluated through electrochemical, morphological, structural, and bendability analyses.

## 2 Experimental Section

### 2.1 Materials and Methods

#### 2.1.1 Preparation of WHCNF

The method used to prepare the WHCNF from water hyacinth fiber pulp is developed based on a previous work,^51^ and is outlined as follows: 60 g of dry water hyacinth powder is soaked in deionized water (DI H_2_O) and homogenized for 15 min at 2000 rpm to form a fiber slurry. The DI H_2_O is removed by using a 5 μm pore size nylon cloth, and the fibers are soaked in sodium hypochlorite and DI H_2_O solution at a 1:3 volume ratio using acetic acid to adjust the pH level to 4. The solution is stirred at 200 rpm and left overnight in a fume hood at 25 °C. The fibers are then washed and filtered using DI H_2_O and dried in the fume hood for 2 h, three times. Next, the fibers are soaked in a sodium hydroxide solution for 2 h, washed in DI H_2_O, filtered with a nylon cloth, and dried in the fume hood three times. Then, fibers are soaked in sodium hypochlorite and DI H_2_O 1:3 volume ratio (acetic acid for adjusting the pH to 4) for 2 h. After washing, filtering using the nylon cloth, and drying the fibers in the fume hood, three more times, the final step is to soak the fibers in DI H_2_O and through a microfluidizer using a 200 μm chamber (10 passes) to make the final WHCNF suspension.

#### 2.1.2 Preparation of the Bilayer Separator

To fabricate the base layer of the separator, 0.15 g (dry weight) of a mixture containing 99 wt % WHCNF and 1 wt % PEG (APC pure, PEG4000) is combined with 10 g of DI H_2_O in a beaker to aid with mixing and dissolving the PEG. The resulting solution is mixed using a T25 digital ULTRA TURRAX for 5 min at 7500 rpm and then poured into a 15 cm diameter Petri dish. To achieve cross-linking, the resulting mixture is freeze–thawed at −20 and 25 °C and then placed in a Gallenhamp vacuum oven at 60 °C for 24 h. The second layer is made of pure PVA (Sigma-Aldrich 363065–500G), which is dissolved in DI H_2_O at 95 °C for 2 h to obtain a 6% PVA concentrated solution. The PVA solution is then doctor-bladed on top of the base layer at a thickness of 150 μm, and the resulting bilayer separators are immersed in an ethanol (Alfa Aesar, 99% pure) bath (NIPS). The bilayer separator is then dried for 3 days at 25 °C in a fume hood and for an additional 24 h in Binder air over at 60 °C, resulting in a ∼37 μm bilayer separator as shown in Figure 1a,b.

### 2.2 Fabrication of Supercapacitors

The primary and secondary paper SCs are fabricated using a MnO_2_/carbon black composite as the positive electrode and zinc metal/carbon black composite as the negative electrode. For these electrodes, two separate composite pastes are prepared. For the primary SC cathode, MnO_2_ (Sigma-Aldrich, <10 μm) was mixed with carbon black (Graphene supermarket, 100 g) and ethyl cellulose (Sigma-Aldrich) as a binder at a weight ratio of 7:1.5:1.5 and terpineol (Sigma-Aldrich) as the solvent. For the secondary SC, the weight ratio of MnO_2_ (Mn) to carbon black (CB) changed to 8:2 with the addition of 40 wt % ethyl cellulose (0.4 g). Similarly, the anode paste is prepared by mixing zinc metals (Sigma-Aldrich, <250 μm) with CB at a weight ratio of 6:4 with 0.4 g of ethyl cellulose; more details and two-dimensional (2D) schematic illustration of the primary and secondary paper SC are shown in Figure 1c,d. For both primary SC composites, a uniform paste is prepared by magnetic stirring for 12 h at room temperature (25 °C). After preparing the paste, the electrode was printed via a doctor blade method on top of the paper substrate (Whatman cellulose filter paper) with a dimension of 2 cm length by 1 cm width (active area 1 cm by 1 cm). The paste is heat-treated at 80 °C for 2 h in the oven. A copper wire is attached for external connection using conductive ink made of silver (Ag) from RS components (186–3600) and dried for 20 min in an air oven at 60 °C. An insulating layer (TPU Protective Ink, JE Solution) is applied on top of the silver conductive ink and dried for 30 min in an air oven at 80 °C. 3 M KOH is prepared as an electrolyte for the SC by dissolving KOH into DI H_2_O (higher molar KOH has a negative effect on the paper electrode as can be seen in Figure S1). The same process is used for the secondary SC, but with the addition of an ∼10-μm-thick silver conductive ink layer as a current collector and a 1 cm × 1 cm dimension of electrode paste on top of the silver conductive ink. Figure 1e shows the schematic diagram of the paper SC and Figure 1f,g shows the Mn/CB-based paper SC (thickness of ∼339 μm) and its bendability.

### 2.3 Characterization

The TMG universal benchtop material thickness gauge, which conforms to ASTM/DIN/EN/ISO standards, is used to measure the thickness of the separators at room temperature. The surface and cross-sectional characteristics of the pristine bilayer separator are observed using a scanning electron microscope (SEM, S4800, Hitachi Company) with an acceleration voltage of 3 kV. The melting point of the separator is examined by differential scanning calorimetry (TA DSC Q2000) from 115 to 175 °C for the Celgard trilayer separator and from 200 to 240 °C for the bilayer separator with a heating rate of 10 °C min^–1^ under nitrogen atmosphere. To study crystal structures of the positive and negative electrodes, the X-ray diffraction (XRD) method is used and tested for the full electrode. It was recorded using a Cu LFF Empyrean tube on the Malvern Panalytical Empyrean system, with a voltage of 45 kV, a current of 40 mA, and a 2θ range of 5–80°. The contact angles of the separators with DI H_2_O and 3 M KOH are determined by taking the mean angle of both the left and right sides of the 5 μL (area) droplet using a contact angle goniometer equipped with image capture (Ossila contact angle Goniometer L2004A1) at room temperature. The air permeability of the separators is assessed by the industry-standard Gurley Densometers Permeability/Porosity 4190N + 4320 model (Gurley time in seconds 100 cm^–3^). The electrolyte uptake (EU) is determined by soaking the separator in a 3 M KOH electrolyte for 4 h at room temperature. Then, eq 1 is used to calculate the EU^29^
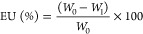
1where *W*_0_ represents the initial mass of the separator before soaking, and *W*_1_ refers to the final mass of the separator after soaking and removing any excess electrolyte from the surface of the separators. The porosity of the separators is determined by soaking them in mineral oil for 4 h at room temperature. Then, eq 2 is used to remove excess mineral oil from the surface of the separator^29^
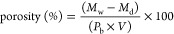
2where *M*_w_ represents the mass of the wet separator, *M*_d_ is the mass of the dry separator, *P*_b_ refers to the density of the mineral oil, and *V* represents the volume of the dry separator. The thermal stability of the separators is evaluated by exposing the ∼1 by 1 cm separator to a temperature ranging from 50 to 150 °C for 1 h in a Binder drying oven. Thermal shrinkage of the separators is investigated by treating them under various temperatures for an hour and then measuring their dimensional changes to determine the thermal shrinkage. The thermal shrinkage is calculated using eq 3(54)
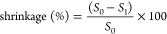
3where *S*_0_ and *S*_1_ represent the area of the separator before after heat treatment, respectively. The ionic conductivity of the separator is measured through an electrochemical system (Ametek Modulab electrochemical system). The separators are soaked in 3 M KOH for 2 h and then inserted between two steel plates to measure the ionic conductivity over a frequency range of 100 Hz to 1 MHz with a signal amplitude of 10 mV. The ionic conductivity (σ) is determined using eq 4(29)
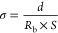
4where *d* represents the thickness of the separators, *R*_b_ represents the bulk resistance, and *S* is the area of the symmetrical electrodes. The electrochemical impedance spectroscopy (EIS) measurement with paper supercapacitors is carried out at 10 mV over a frequency spectrum spanning from 1 Hz to 1 MHz with a sinusoidal signal of 10 mV. Cyclic voltammetry (CV) analysis was carried out at a scan rate of 5–200 mV s^–1^ in a potential range between 0 and 0.6 V. The Coulombic efficiency of the paper SCs with the separators while being charged and discharged at 700–1000 μA discharge current from 0 to 0.6 V is calculated using eq 5
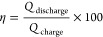
5where *Q* discharge represents the charge in coulombs during discharging, and *Q* charge represents the charge in coulombs while the supercapacitor is charging. The specific capacitance of the paper supercapacitors with the separators at various galvanostatic charge–discharge rates, ranging from 700 to 1000 μA, is calculated using eq 6
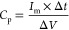
6where *I*_m_ represents the current density, Δ*t* represents the discharge time taken by the paper SCs, and ΔV represents the discharge voltage drop measured from galvanostatic charging–discharging (GCD) analysis. The energy and power densities of paper SCs are calculated using eqs 7 and 8
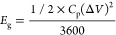
7where the *C*_p_ value is computed by eq 6, and Δ*V* is the squared value of the discharge voltage drop
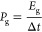
8For power density, the *E*_g_ value is computed by eq 7, and Δ*t* represents the discharge time taken.

## 3 Results and Discussion

### 3.1 Morphology and Structural Characterization

**Figure 2:**
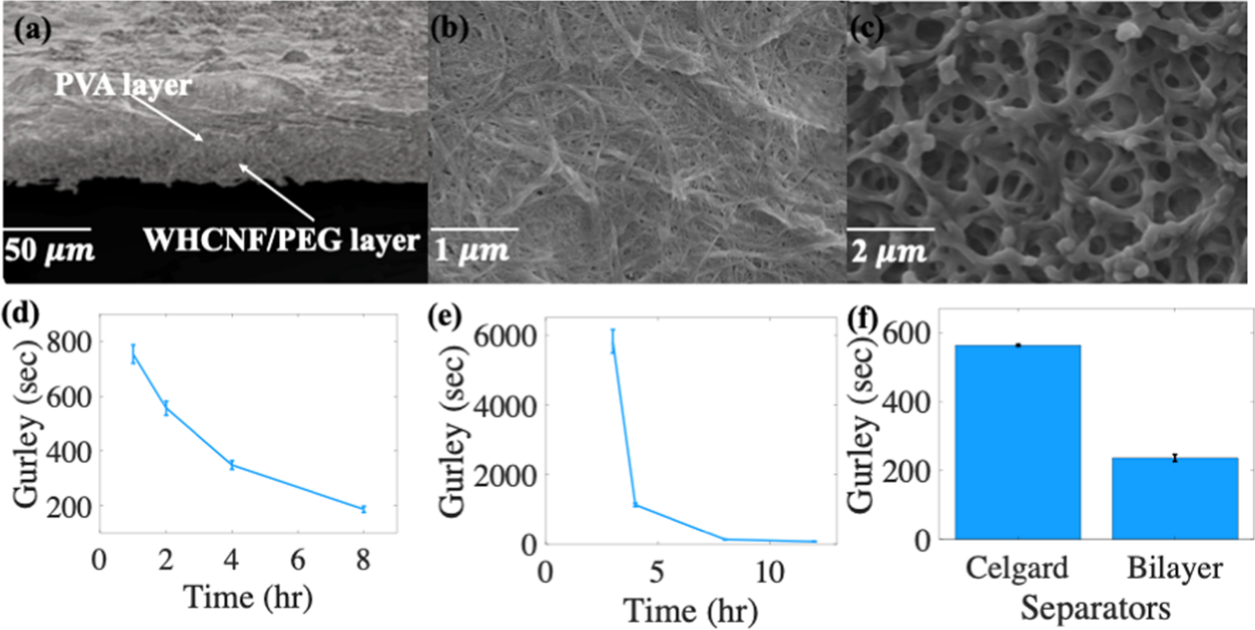
(a) SEM image of the cross-sectional area of the bilayer separator at 1K magnification, (b) SEM image of the WHCNF/PEG layer of bilayer separator (×15k magnification), and (c) SEM image of the PVA layer on top of the WHCNF/PEG layer at a magnification of 1k. (d–f) Air permeability of the WHCNF/PEG layer during the freeze–thawing process, the PVA layer in ethanol, and the bilayer and Celgard separators, respectively.

Figure 2a represents the cross-sectional area of the designed separator. The bottom half of the separator (∼20 μm thick) is made of 99% WHCNF and 1% PEG as a cross-linker using the freeze–thawing method to enhance flexibility, wettability, and electrolyte uptake. The top of the separator is then coated with pure PVA at a thickness of 150 μm by a doctor blade, resulting in a ∼20-μm-thick PVA layer. This layer is utilized for the high-temperature separator shutdown effect at ∼230 °C, via the nonsolvent-induced phase separation method. The surface morphology of the WHCNF/PEG layer shows the cellulose fibers and their porous structure as shown in Figure 2b. The 3D interconnected PVA layer porous structure on top of the WHCNF/PEG layer is shown in Figure 2c. Figure 2d demonstrates the impact of the time spent in the freeze–thaw method on the WHCNF/PEG layer air permeability. When frozen for 1 h and thawed for 1 h at the temperatures of −20 and 25 °C, the mean Gurley seconds are 753.9 (Table S2), which reduces further with the more time spent freeze–thawing. The WHCNF/PEG layer used in this study was subjected to 8 h of the freeze–thawing process, resulting in a mean air permeability of 186.9 Gurley seconds. Regarding the pure PVA layer, Figure 2e, individual PVA film layers are fabricated to test their air permeability before doctor-blading on top of the WHCNF/PEG layer. After spending 12 h in ethanol during the nonsolvent-induced phase separation method, the mean air permeability is 73 Gurley seconds (Table S3). However, leaving it in ethanol for longer than 12 h resulted in the film becoming stiff and rigid (Figure S2). The resulting bilayer separator, consisting of 8 h of freeze–thawing and 12 h of the nonsolvent-induced phase separation method, demonstrates a total mean air permeability of 236.7 Gurley seconds (Table S4). This is in comparison to the commercially available Celgard 2320 separator, which shows a mean air permeability of 563.4 Gurley seconds, as shown in Figure 2f.

**Figure 3:**
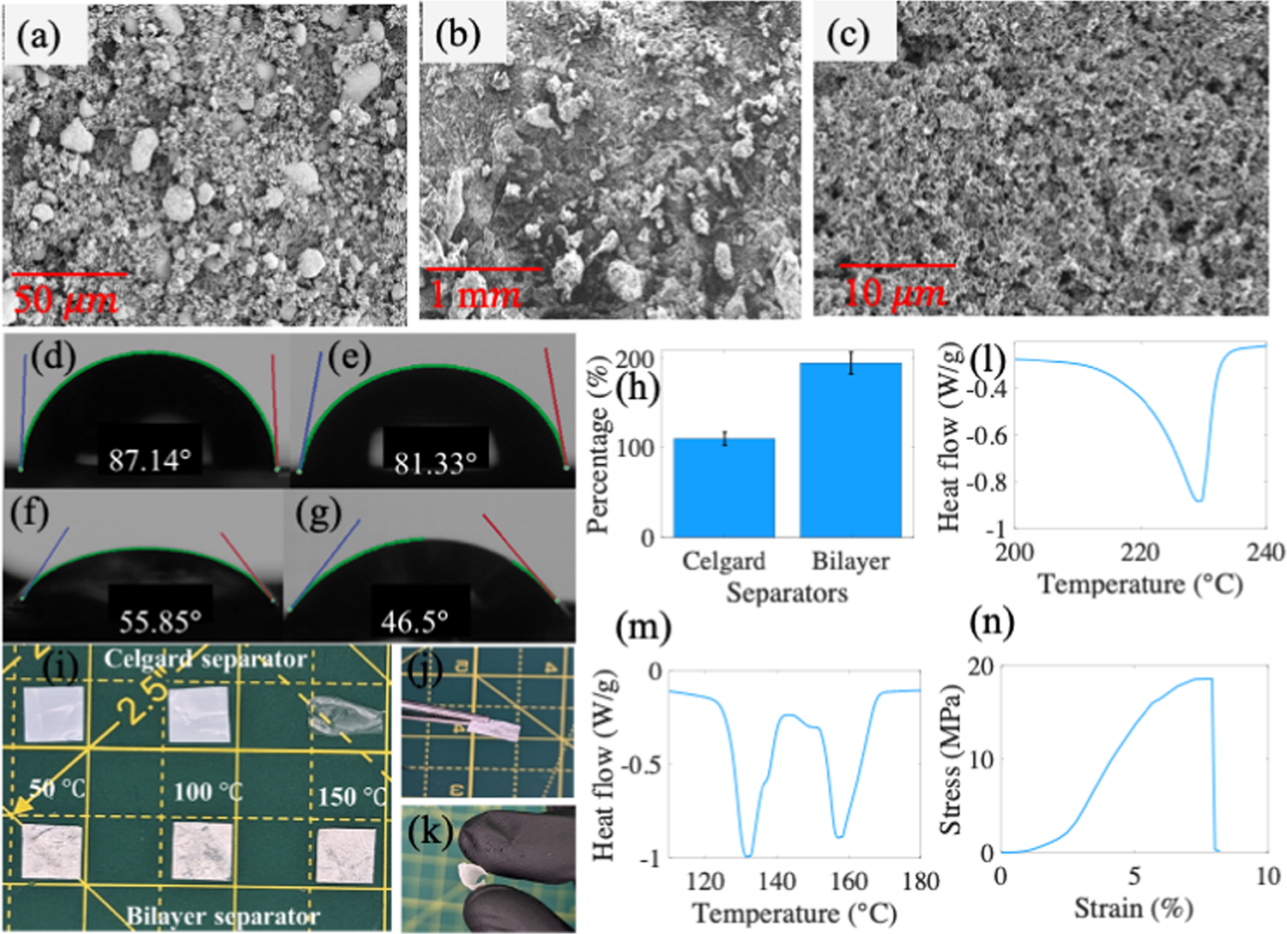
Morphology of primary SC (a) Mn/CB and (b) Zn metal/CB electrodes at a magnification of ×800 (c) SEM image of binder-rich CB used for the secondary paper electrode. (d, e) Contact angle measurement by using DI H_2_O of Celgard and bilayer separators. (f, g) Contact angle measurement using 3 M KOH of Celgard and bilayer separator, respectively. (h) Electrolyte uptake percentage of Celgard and bilayer separators. (i) Photograph of the thermal shrinkage of Celgard and bilayer separators. (j) Bilayer separator used with the SC. panel (k) demonstrates its flexibility and bendability. (l, m) DSC results from the bilayer and celgard separator, respectively. (n) Bilayer separators’ tensile strength of 18 MPa.

In addition, the morphology of Mn/CB and zinc metal/CB composite electrodes is investigated as shown in Figure 3a,b; the SEM images of the Mn electrode (Mn particle size <10 μm) are more abundant and more evenly distributed on the surface than those of the Zn metal (Zn metal particle size <250 μm), which are used to increase the electrical conductivity of the anode, respectively. The loosely bonded particles in the electrodes are clearer to see at 50 μm (Figure 3a), less amount of binder (ethyl cellulose) is used for the fabrication of the primary paper SC, and Figure 3c shows the SEM image of the Mn electrode in the secondary paper SC.

Table 1 displays the thickness, wettability, EU, air permeability, and porosity of commercial Celgard 2320 and bilayer separators. Due to the high porosity, hydrophilicity, and good interfacial compatibility with electrodes, separators provide a superior EU and low interfacial resistance, resulting in enhanced ionic conductivity.^36^ As illustrated in Table 1, the bilayer separator exhibits a higher porosity and air permeability (46%, 236.7 Gurley seconds) compared to Celgard, which has a porosity of 39% and air permeability of 563.4 Gurley seconds. This leads to a higher EU of 194% compared to 110% for Celgard. Additionally, the bilayer separator displays a lower DI H_2_O contact angle of 81.33° compared with Celgard at 87.14°, showing the more hydrophilic nature of the separator.

**Table 1:** Thickness, Wettability, EU, Air Permeability, and Porosity Values of Celgard 2320 and the Bilayer Separator

separators	thickness (μm)	wettability (deg)	EU (%)	air permeability (Gurley seconds)	porosity (%)
Celgard 2320	20	87.14	110	563.4	39
bilayer	37	81.33	194	236.7	46

### 3.2 Wettability and Electrolyte Uptake

To assess the wettability of the Celgard and bilayer separators, contact angle measurements are taken and compared using DI H_2_O and 3 M KOH. Figure 3d shows that the mean contact angle of Celgard when using DI H_2_O is 87.14°, while the bilayer separator exhibits a mean contact angle of 81.33° shown in Figure 3e; more details can be seen in Table S5. Similar results are observed when using 3 M KOH electrolyte solution in Figure 3f,g, resulting in a mean contact angle of 55.85 and 46.5° for Celgard and bilayer separators, respectively. Both sets of results indicate that the bilayer separator demonstrates higher wettability than the Celgard separator. The higher porosity and the use of hydroxyl group-based materials, such as WHCNF, PEG, and PVA, to fabricate the bilayer separator contributes to the hydrophilic nature of the separator and a strong affinity with water and polar electrolytes, which contributes to its ability to readily absorb liquids and decrease internal resistance of the energy storage device.

To further investigate the electrolyte performance of the Celgard and bilayer separator, the electrolyte uptake is studied, and the findings are presented in Tables 1 and S6, and Figure 3h. The results indicate that the electrolyte uptake of the bilayer separator is higher than that of the Celgard separator. This can be attributed to the higher porosity, wettability, and air permeability of the bilayer separator, which are expected to enhance its efficiency and ionic conductivity and reduce internal resistance in paper SC.

### 3.3 Thermal Stability and Shutdown Effect

Thermal stability is crucial for separators, as high temperatures can cause shrinking of the separator that can result in the electrodes contacting each other, leading to a short circuit and thermal runaway.^55^Figure 3i examines the performance of Celgard and bilayer separators at temperatures of 50, 100, and 150 °C. The results indicate the Celgard experiences significant thermal shrinkage of ∼50% at 150 °C, while the bilayer separator is more stable, with shrinkage of approximately ∼5%. Hence, it can be concluded that the bilayer separator is superior in terms of thermal resistance and provides a higher resistance to short circuits. Figure 3j,k shows a close-up view of a 1 cm^2^ bilayer separator utilized in the paper supercapacitor and demonstrates its bendability.

To further investigate the thermal properties of separators and the separator shutdown effect, Celgard and bilayer separators are subjected to differential scanning calorimetry (DSC). The shutdown effect is a crucial property of separators, wherein high temperatures cause the separator to melt, closing its pores and halting the electrochemical device’s function.^55^ Celgard is composed of two types of polymers, with PP sandwiched between two layers of PE. As shown in Figure 3m, the PE melting temperature is ∼132 °C, and the PP melting temperature is ∼157 °C, which can explain the shrinkage of the Celgard separator at 150 °C. However, the bilayer separator’s layer two comprises PVA, which aids in assisting in the shutdown effect and has a melting point of ∼228 °C, as illustrated in Figure 3l. This characteristic indicates greater heat resistance and beneficial impact on thermal stability. The tensile strength of the bilayer separator is also measured to be 18 MPa, as shown in Figure 3n.

### 3.4 Electrochemical Properties of Bilayer Separators and the Crystal Structure of Electrode Materials

**Figure 4:**
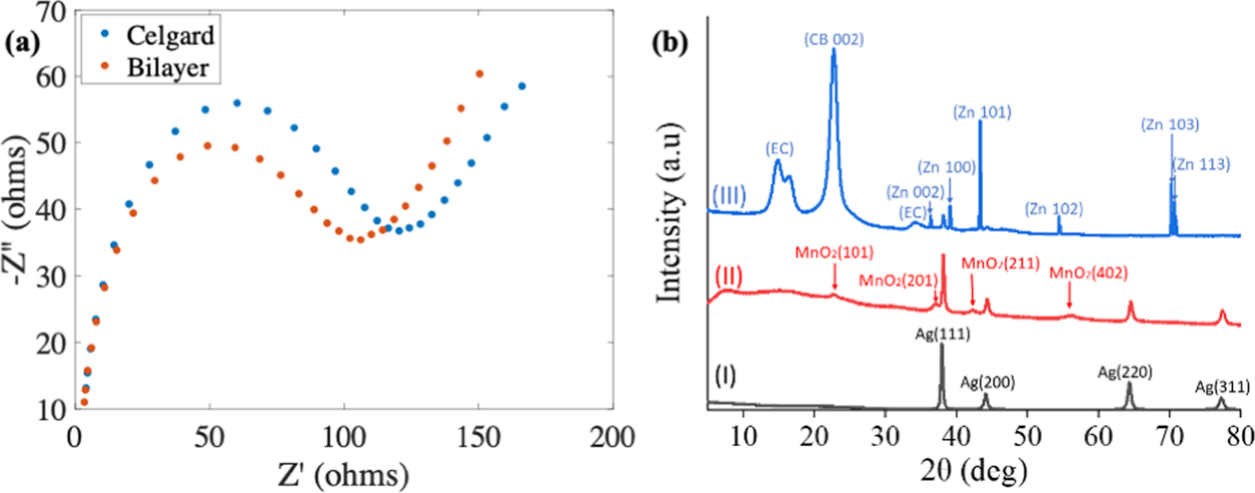
(a) Nyquist plot of the SS/separator-3 M KOH/SS cell with Celgard and bilayer separators and (b) XRD pattern of the printed electrode.

EIS analysis is carried out to assess the ionic conductivity of the separators. This involves soaking both separators in a 3 M KOH electrolyte between two stainless steel (SS) electrodes.^20^Figure 4 shows the Nyquist plot for both Celgard and bilayer separators. The intercept of the Nyquist plot on the *Z*_real_ axis represents the bulk resistance (*R*_b_), which is used with eq 4 to get the ionic conductivity of the separators.^29^ The *R*_b_ values for the Celgard and bilayer separators are ∼1.8 and ∼1.4 Ω, respectively. The ionic conductivity of Celgard is calculated to be 0.22 mS cm^–1^, while the bilayer separator has an ionic conductivity of 0.52 mS cm^–1^. The higher ionic conductivity of the bilayer separator can be attributed to its high porosity that allows for more flow of ions through the separator. Additionally, its superior wettability and electrolyte uptake facilitate a reduction in internal resistance and more storage of the ions in the separators.^29^Figure 4b(I) shows the XRD pattern of carbon black (CB) and ethyl cellulose (EC) on silver conductive ink coated on the paper substrate. It exhibits four main characteristic peaks associated with a silver cubic (FCC) crystal structure at 2θ angles of 28.2, 44.3, 64.5, and 77.5° corresponding to the (111), (200), (220), and (311) planes.^56^ The broad diffraction 2θ region (∼5–28°) can be attributed to the amorphous structures of ethyl cellulose and carbon black. In Figure 4b(II), the cathode material is a doctor blade on top of the silver conductive ink current collector. The silver peaks are still visible, along with the addition of γ-MnO_2_ peaks and their corresponding 2θ angles and planes. The γ-MnO_2_ crystalline structure consists of a combination of 1 × 1 and 1 × 2 tunnels with interlayer separations of 1.89 and 2.3 Å, which is the same structure used in positive electrodes, such as electrolytic manganese dioxide for alkaline batteries.^57,58^ In Figure 4b(III), the anode material is coated on top of the silver current collector. Ethyl cellulose and carbon black exhibit more defined amorphous peaks when compared to those of the cathode material. The diffraction peaks of the hexagonal closed pack structure of Zn are shown at 2θ angles of 36.34, 39.06, 43.28, 54.36, 70.1, and 70.68° corresponding to the (002), (100), (101), (102), (103), and (113) planes.^59^

### 3.5 Paper-Based Mn/CB and Zn Metal/CB Supercapacitors

**Figure 5:**
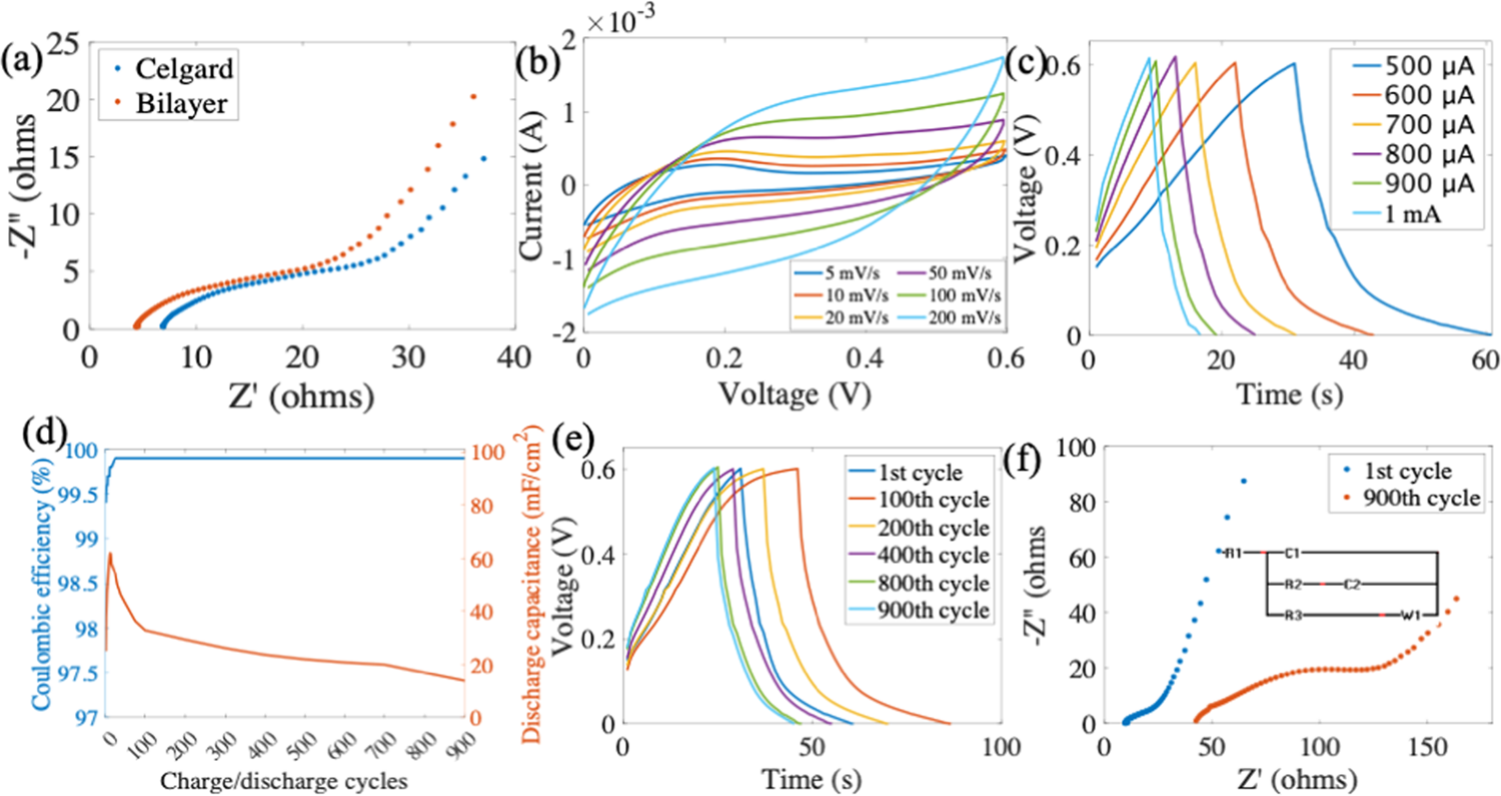
(a) EIS of the Mn/CB paper secondary SC with Celgard and bilayer separators, panels (b, c) show the CV and GCD of the secondary SC with the bilayer separator, (d) Coulombic efficiency and discharge capacitance of 900 cycles of the SC with the bilayer separator, (e) GCD curves for various cycles of secondary SC with the bilayer separator. (f) EIS of the 1st and 900th cycle and the 1st cycle’s equivalent circuit for the secondary SC with the bilayer separator.

To evaluate the performance of bilayer separators in energy storage, two devices are fabricated and compared: primary and secondary paper SC. The electrochemical properties of the primary SC (schematic representation in Figure S3) were analyzed for the bilayer separator. The electrochemical performance of this SC is given in Figure S4. The primary SC without the silver conductive ink showed inconsistent results with the ohmic resistance ranging from 89.17 to 6032.01 Ω, and the best resulting SC without the silver conductive ink data is shown in Figure S4. The interfacial compatibility of separators with the Mn/CB with Zn metal/CB electrode is assessed by using the fully fabricated secondary SC shown in the schematic representation in Figure S5 to compare the Celgard and bilayer separator performance. Figure 5a shows the Nyquist plot (frequency range of 1 Hz to 1 MHz) of Mn/CB with 3 M KOH. The *Z*′ (*Z*_real_) axis intercept indicates the ohmic resistance (*R*_s_), while the semicircle at a high-frequency range corresponds to the interfacial resistance between the separator and electrodes (*R*_ct_). The *R*_s_ values for Celgard and bilayer separators are 6.95 and 4.57 Ω, respectively. Also, the bilayer separator has a smaller semicircle, representing an improved *R*_ct_. Both electrochemical resistances show that the bilayer separator has lower resistances than the Celgard, indicating more efficient ion transportation between the bilayer separator and the electrodes. This can be attributed to the bilayer separator’s higher porosity and better electrolyte wettability.^27^ CV analysis with a bilayer separator was carried out to analyze the electrochemical reaction. The pseudocapacitance of the electrodes contributes to enhancing the performance of the paper SC, as shown in the CV; see Figure 5b at various scan rates (5–200 mV s^–1^). The presence of carbon black contributes to electrochemical double-layer formation, and MnO_2_ enhances the pseudocapacitance of the electrodes, resulting in the CV curve of the paper SC exhibiting a quasi-rectangular behavior.

Furthermore, GCD analysis was carried out to investigate the energy-storing performances. Figure 5c presents GCD at 500 μA to 1 mA for SC with the bilayer separator; the energy and power density of the SC are calculated from GCD analysis shown in Table S7 (the data for the 500 uA is collected from the 100th cycle when the cell discharge capacitance is more stable, as seen in Figure 5d). The paper secondary SC shows a specific capacitance of 34.1 mF cm^–2^ and energy and power density of 1.07 μWh cm^–2^ and 204.8 μW cm^–2^ at 500 μA, respectively. The efficiency remained constant throughout the 900 cycles, gradually increasing to 99.9% efficiency after the initial 25 cycles as shown in Figure 5d. The GCD (Figure 5e) for each cycle exhibits a similar pattern to the discharge capacitance, being initially inconsistent and stabilizing from 100th cycle onward. The discharge capacitance (mF cm^–2^) dropped from 34.1 for the 100th cycle to 18.3 for the 900th cycle, representing a decrease of 46.3%. The EIS (Figure 5f) shows an increase in *R*_s_ and *R*_ct_ from the initial cycle to the 900th cycle, with the *R*_s_ values for the first and 900th cycle being 9.7 and 41.8 Ω, respectively. The inset in Figure 5f shows the equivalent circuit with Warburg impedance for the first cycle of the paper SC, which can be seen more clearly in Figure S6, along with the values of each component in the equivalent circuit (Table S8).

**Figure 6:**
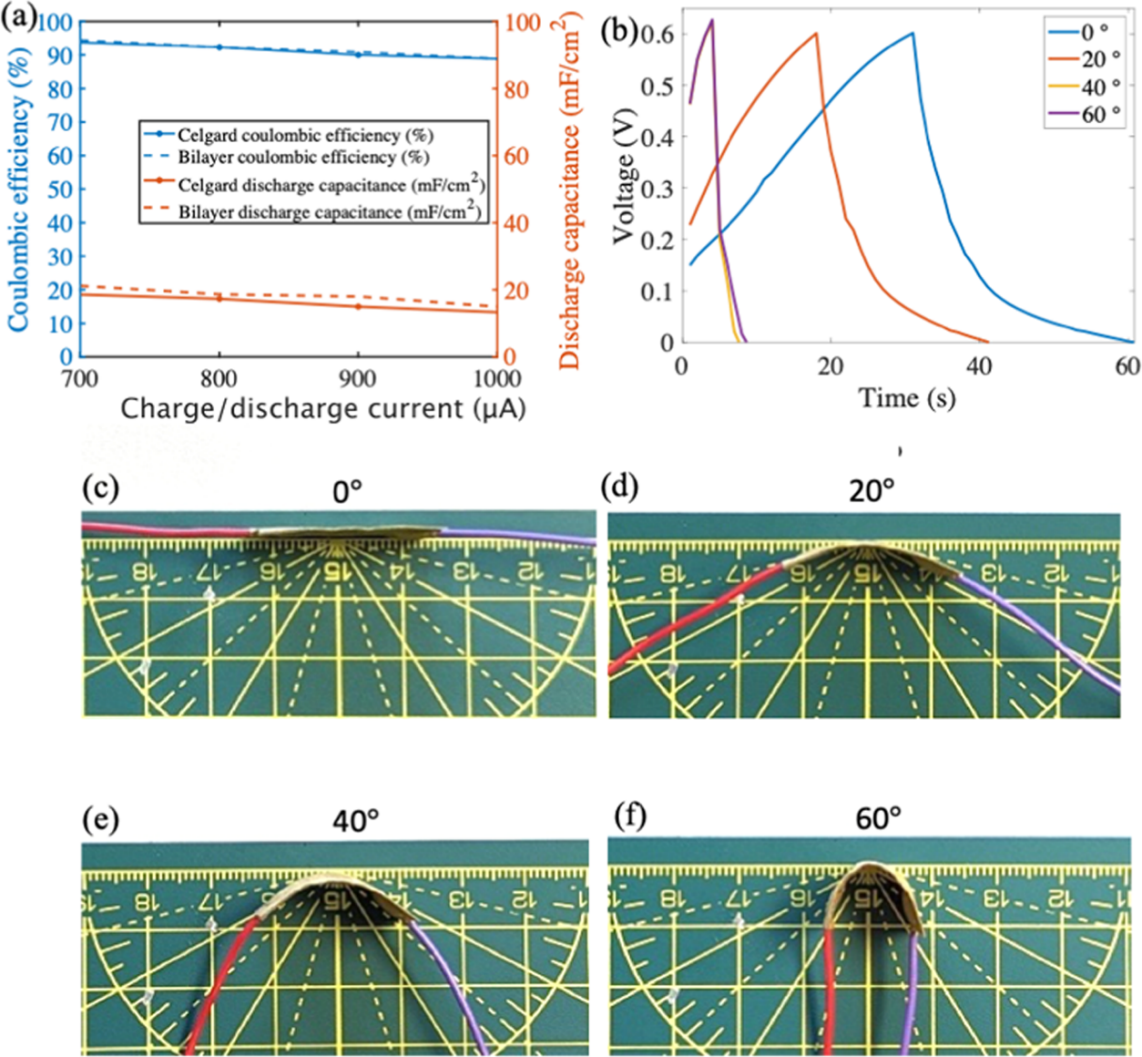
(a) Efficiency and discharge capacitance at the charge/discharge current from 700 to 1000 μA. (b) GCD curve of paper SC with the bilayer separator under different bending angles and panels (c–f) show paper SC flexibility under varying degrees of bending.

Figure 6a and Table S9compare the Coulombic efficiency and discharge capacitance of the Mn/CB paper supercapacitor at various charge/discharge rates ranging from 700 to 1000 μA, and from 0 to 0.6 V, the GCD of the SC with the Celgard and Bilayer separators are shown in Figure S7. The bilayer separator exhibits a higher mean efficiency of 0.44% than the Celgard across charge/discharge rates of 700, 800, 900, and 1000 μA as shown in Figure 6a. Additionally, the bilayer separator shows better mean discharge capacitance (mF cm^–2^), a higher mean discharge capacitance of 13.53% than the Celgard for the paper SC. The superior efficiency and discharge capacitance can be attributed to the bilayer separator’s higher porosity, excellent electrolyte uptake, and electrolyte wettability, which also increases the ionic conductivity.^27^

To evaluate the performance of the paper SC bendability with the bilayer separator, the paper-based SC’s bendability is analyzed by subjecting the device to various degrees of bending while charging and discharging (GCD) at 500 μA, as shown in Figure 6b and documented in Table S10. The bendability is found to have an impact on the device, with a discharge capacitance retention of 80.6% at 20°. Figure 6c–f shows paper SC flexibility under varying degrees of bending.

## 4 Conclusions

In summary, water hyacinth fiber pulp is used to prepare WHCNF, which are then utilized to fabricate a bilayer separator using eco-friendly materials through a two-step process of freeze–thawing and nonsolvent-induced phase separation. A complete investigation, including morphology, wettability, electrolyte uptake, porosity, thermal stability, and cell performance, is carried out by fabricating a paper SC. The bilayer separator demonstrates superior properties compared to the Celgard separators, including higher porosity (46%), improved wettability (46.5°) with 3 M KOH electrolyte, and electrolyte uptake (194%), resulting in high ionic conductivity (0.52 mS cm^–1^). The bilayer separator is thermally stable at temperatures up to 150 °C. Additionally, the bilayer separator exhibits a higher mean efficiency of 0.44% than Celgard across GCD rates of 700, 800, 900, and 1000 μA, and mean discharge capacitance 13.53% higher than the Celgard separator for the paper supercapacitor. Furthermore, a flexible and thin (∼339 μm) paper SC is fabricated with the incorporation of the Zn metal to increase the anode electrical conductivity. The paper supercapacitor demonstrates a specific capacitance of 34.1 mF cm^–2^ and energy and power density of 1.70 μWh cm^–2^ and 204.8 μW cm^–2^ at 500 μA, respectively. The bendability analysis shows that the paper supercapacitor retains 80.6% of its discharge capacitance retention at 20°. This work presents an eco-friendly new bilayer separator and Mn/CB-based paper supercapacitors, providing a sustainable alternative to petroleum-based polymer separators and toxic materials commonly used in electrochemical energy-storage devices.

## Supporting Information Available

Details of performances of supercapacitors and properties of the prepared materials and electrodes (PDF)
