# Supplementary material for: Paper Supercapacitor Developed Using a Manganese Dioxide/Carbon Black Composite and a Water Hyacinth Cellulose Nanofiber-Based Bilayer Separator
Source: ACS Appl Mater Interfaces. 2023 Oct 28;15(44):51100–9. doi: 10.1021/acsami.3c11005 (PMC10636709; doi:10.1021/acsami.3c11005)
Supplement: Supplementary file 1 — am3c11005_si_001.pdf [file am3c11005_si_001.pdf]

Supporting Information

Paper Supercapacitor Developed Using Manganese Dioxide /Carbon Black Composite and Water Hyacinth Cellulose Nanofiber-based Bilayer Separator

Mustehsan Beg, Keith M. Alcock, Achu Titus Mavelil, Dominic O'Rourke, Dongyang Sun, Keng Goh, Libu Manjakkal*, and Hongnian Yu*

School of Computing and Engineering & the Built Environment, Edinburgh Napier University, Merchiston Campus, EH10 5DT, UK.

Email: H.Yu@napier.ac.uk; Li.Manjakkal@napier.ac.uk

Table S1: Performance of paper-based supercapacitors.

Type of electrode	Electrolyte	Specific capacitance	Energy density	Power density	References
PEDOT: PSS-CNF	HEC: EMIM-ES	5.2 mF cm^{-2}	0.27 μWh cm^{-2}	10.5 mW cm^{-2}	1
Graphite-AC-CNC	NaCl-gly-CNC	25 mF cm^{-2}	0.88 Wh kg^{-1}	830 W kg^{-1}	2
TREN/TOABr-Au NP	1 M Na_2SO_4	1.35 mF cm^{-2}	267.3 µW h cm^{-2}	128.9 kW kg^{-1}	3
CNT-BNC	CoPolymer-EMIM NTf_2 C-TRI	20 mF cm^{-2}	15.5mWh g^{-1}	1.5W g^{-1}	4
rGO/cellulose paper	1 M Na_2SO_4	24 mF cm^{-2}	24.6 Wh kg^{-1}	503 W kg^{-1}	5
Graphite /activated carbon /CNF/CNC	CNC/glycerol/NaCl	25 mF cm^{-2} 25.6 F g^{-1}	0.88 Wh kg $^{-1}$	830 W kg $^{-1}$	6
PEDOT paper	PVA/H_2SO_4	13.7 F/cm^3	0.76 mWh/cm^3	0.01 W/cm^3	7
BNNT–CNF/ZnO	3 M KOH	94 F g^{-1}	30.3 W h kg^{-1}	1.3 kW kg^{-1}	8
bismuth ferrite/graphene	1 M Na_2SO_4	9 mF/cm^2	-	0.5–3.5 kW/kg	9
PEDOT:PSS/cellulose nanofibril	PVA–H_2SO_4	-	0.013 Wh/cm^3	1 W/cm^3	10
This research (primary paper supercapacitor)	3 M KOH	25.5 mF cm^{-2}	1.27 μWh cm^{-2}	27 μW cm^{-2}	This work
This research (secondary paper supercapacitor)	3 M KOH	34.1 mF cm^{-2}	1.70 μWh cm^{-2}	204.8 μWcm^{-2}	This work

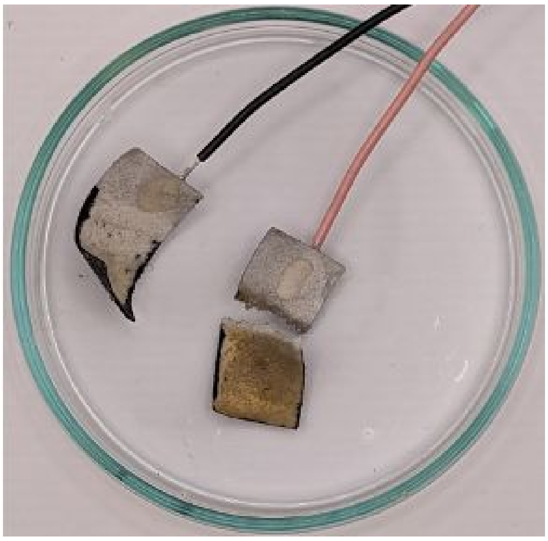

Figure S1: Shows the effect of higher molar KOH on the paper substrate.

Electrical conductivity of Anode

To enhance the electric conductivity of the anode, the anode ink fabrication incorporates Zinc metal particles of the size of < 250 μm. The conductivity (σ) is calculated using the equation:

$$\sigma = \frac{1}{Rt} x \frac{L}{A}$$

Where, Rt is the total resistance of the anode, L and A are the length and cross-sectional area in cm^2. Prior to the addition of Zinc, the anode exhibited an electrical conductivity of 4.85 x 10^{-6} S cm^{-1}. However, after integrating 0.7 g of Zinc, the electrical conductivity increased to 1.45 x 10^{-5} S cm^{-1}, resulting in a percentage increase of ∼195 %.

Materials used for the electrode's development

Primary Cathode - MnO_2– 7 wt. % - 0.7 g. Carbon black – 1.5 wt. % - 0.15 g
Ethyl-cellulose – 1.5 wt. % - 0.15 g
Sol**vent –** Terpineol - 4:1 – 4 g

Secondary Cathode - MnO_2– 8 wt. % - 0.8 g
Carbon black – 2 wt. % - 0.2 g
Ethyl-cellulose – 40 wt. % of total ink - 0.4 g
Solvent – Terpineol - 4:1 – 4 g

Primary Anode - Zinc metal -7 wt. %% – 0.7 g
Carbon Black –1.5 wt.%- 0.15 g
Ethyl-cellulose – 1.5 wt. %– 0.15 g

Secondary Anode - Zinc metal -6 wt. %% – 0.6 g
Carbon Black –4 wt.%- 0.4 g
Ethyl-cellulose – 40 wt. % of total ink– 0.4 g

Table S2: Base layer 99% WHCNF/1% PEG air permeability per freeze-thawing hours.

Layer 1: WHCNF/PEG Hours freeze-thawing	Sample 1	Sample 2	Sample 3	Mean	Standard deviation
**1**	765.6	716.1	780.1	753.9	33.5
**2**	534.2	549.1	585.2	556.1	26.2
**4**	360.2	354.2	330.5	348.3	15.7
**8**	198.3	175.9	186.5	186.9	11.2

Table S3: PVA layer air permeability per hour during NIPS process.

Layer 2: PVA Hours in ethanol	Sample 1	Sample 2	Sample 3	Mean	Standard deviation
**3**	5717.8	6199.4	5552.5	5823.3	336.1
**4**	1112.7	1099.7	1187.2	1133.2	47.2
**8**	136.3	144.1	132.6	137.6	5.87
**12**	70.8	78.6	69.7	73.1	4.8

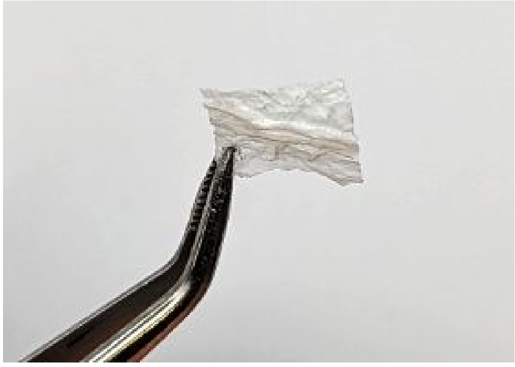

Figure S2: Over 12 hours spend in ethanol results in PVA layer becoming stiff and rigid.

Table S4: The air permeability of Celgard and bilayer separators.

Celgard and bilayer Separators	Sample 1	Sample 2	Sample 3	Mean	Standard deviation
**Celgard**	561.4	566.7	562.3	563.4	2.8
**Bilayer**	239.4	226.1	245.6	236.7	9.9

Table S5: The wettability of Celgard and bilayer separators in both DI H_2O and 3M KOH solutions.

Separators	Left angle (°)	Right angle (°)	Mean angle (°)
**Celgard (DI H_2O)**	86.83	87.44	87.14
**Bilayer (DI H_2O)**	60.16	55.85	58.01
**Celgard (1M KOH)**	81.05	81.33	81.19
**Bilayer (1M KOH)**	47.91	45.08	46.5

Table S6: The electrolyte uptake results of Celgard and bilayer separators.

Separators	Sample 1	Sample 2	Sample 3	Mean angle (°)	Standard deviation
**Celgard**	104	118	108	110	7.21
**Bilayer**	199	180	203	194	12.28

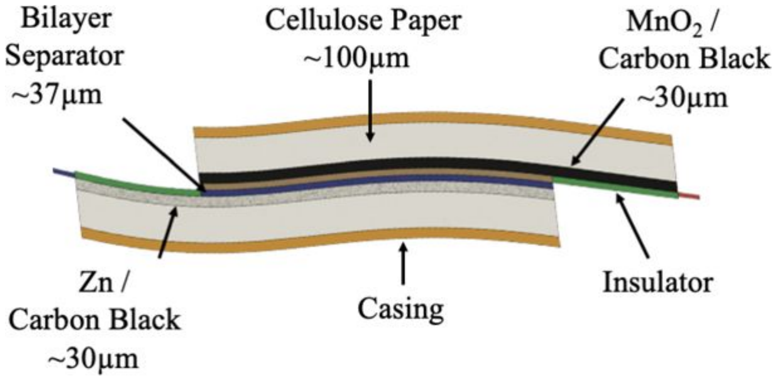

Figure S3: Schematic diagram of Mn /CB primary paper supercapacitor.

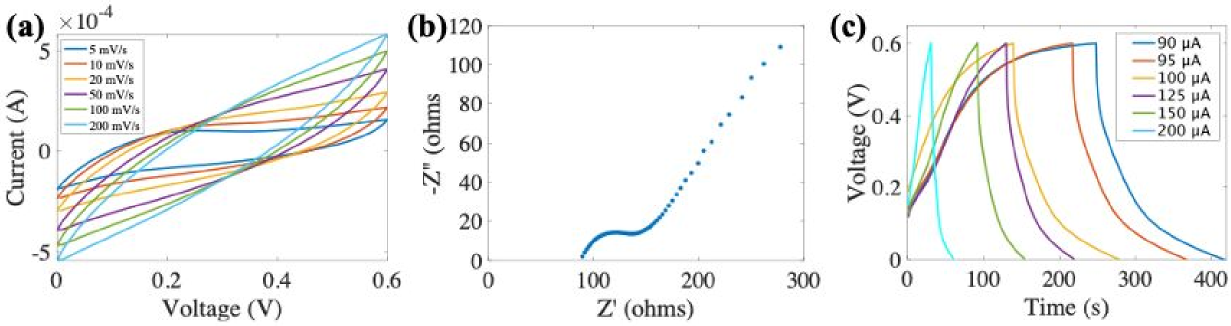

Figure S4: a) The CV from 5-200 mV s^{-1}, b) and c) the EIS and GCD of the paper SC.

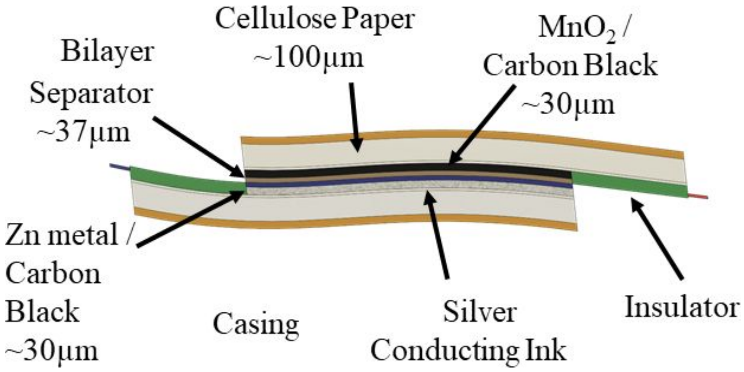

Figure S5: Schematic diagram of Mn /CB secondary paper SC.

Table S7: Discharge capacitance, energy, and power densities of Mn-CB paper SC.

Charge/discharge current (μA)	Discharge capacitance (mF cm^{-2})	Energy density(μWh cm^{-2})	Power density (μW cm^{-2})
500	34.1	1.70	204.8
700	21.16	1.05	201.9
800	18.66	0.93	244.7
900	18	0.9	272.7
1000	15	0.75	300

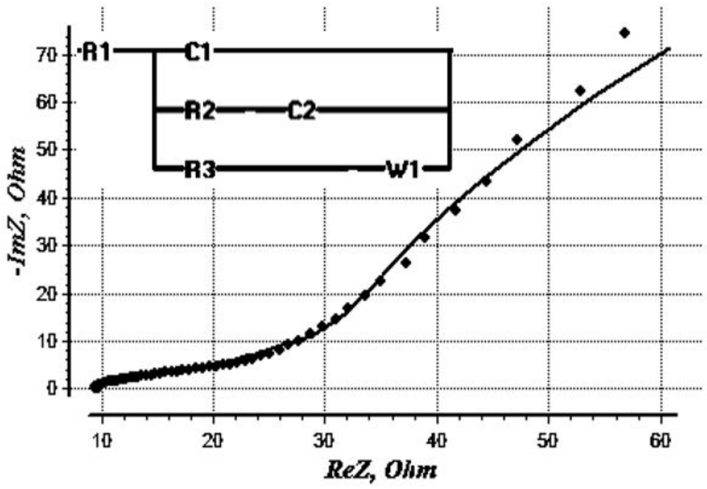

Figure S6: The figure of the accuracy of the fitting (solid line) and insert showing the equivalent circuit.

Table S8: Table of values of each component in the equivalent circuit.

Components	Values
C1	1.6151E-06
C2	0.0003672
R1	10.016
R2	51.852
R3	4.8809
Aw1	339.25

Table S9: Celgard and bilayer separators efficiency and discharge capacitance.

Discharge current (μA)	Celgard coulombic η (%)	Bilayer coulombic η (%)	Celgard discharge capacitance (mF cm^{-2})	Bilayer discharge capacitance (mF cm^{-2})
**700**	93.75	94.44	18.6	21.16
**800**	92.31	92.32	17.3	18.66
**900**	90	90.9	15	18
**1000**	88.88	88.89	13.33	15

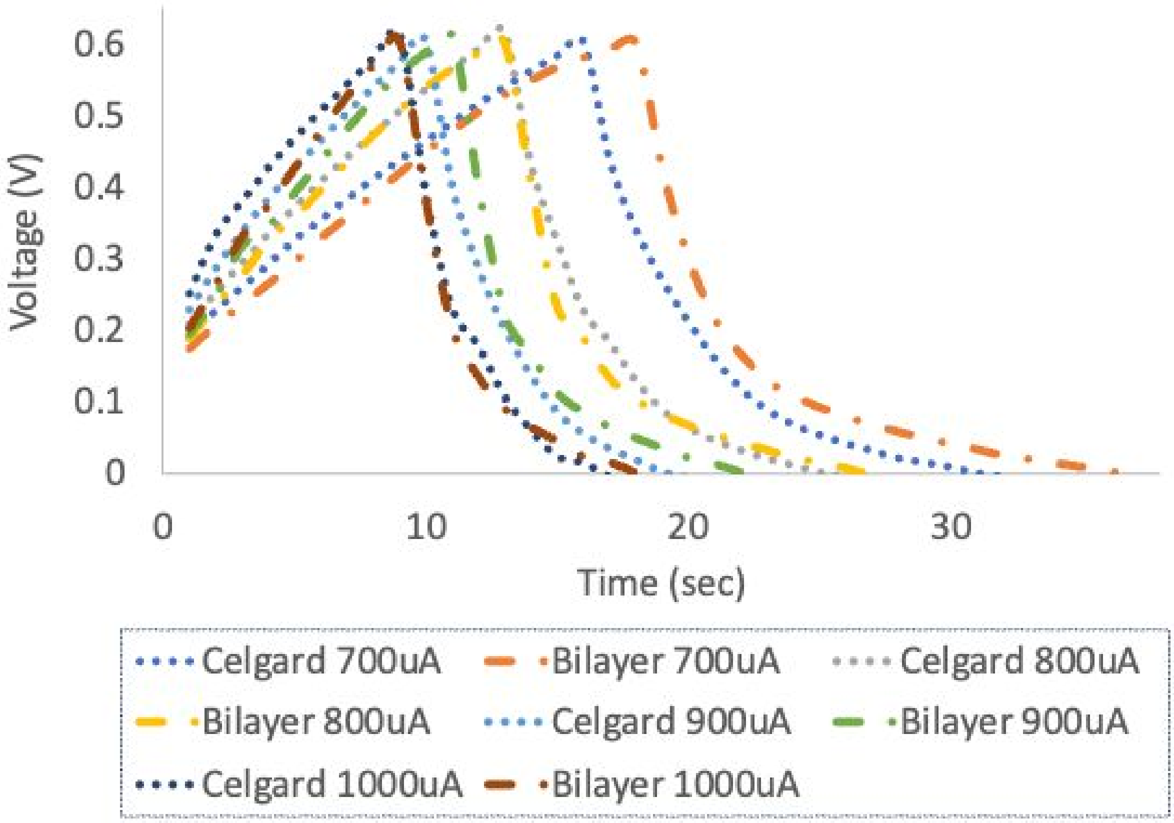

Figure S7: Paper supercapacitor discharge curves.

Table S10: bendability analysis at 500 μA.

Bendability (°)	Coulombic η (%)	Discharge Capacitance (mF cm^{-2})	Energy Density (μ Wh cm^{-2})	Power Density (μ W cm^{-2})	Discharge Capacitance Retention (%)
0	95.4	10.3	0.51	59.3	0
20	94.4	8.3	0.41	59.4	80.6
40	75	1.6	0.08	62.5	15.6
60	74.9	2	0.1	62.5	19.5

References

(1) Say, M. G.; Brooke, R.; Edberg, J.; Grimoldi, A.; Belaineh, D.; Engquist, I.; Berggren, M. Spray-Coated Paper Supercapacitors. *npj Flexible Electronics* **2020**, *4* (1), 14. https://doi.org/10.1038/s41528-020-0079-8.

(2) Aeby, X.; Poulin, A.; Siqueira, G.; Hausmann, M. K.; Nyström, G. Fully 3D Printed and Disposable Paper Supercapacitors. *Advanced Materials* **2021**, *33* (26), 2101328. https://doi.org/10.1002/adma.202101328.

(3) Ko, Y.; Kwon, M.; Bae, W. K.; Lee, B.; Lee, S. W.; Cho, J. Flexible Supercapacitor Electrodes Based on Real Metal-like Cellulose Papers. *Nature Communications 2017 8:1* **2017**, *8* (1), 1–11. https://doi.org/10.1038/s41467-017-00550-3.

(4) Kang, Y. J.; Chun, S. J.; Lee, S. S.; Kim, B. Y.; Kim, J. H.; Chung, H.; Lee, S. Y.; Kim, W. All-Solid-State Flexible Supercapacitors Fabricated with Bacterial Nanocellulose Papers, Carbon Nanotubes, and Triblock-Copolymer Ion Gels. *ACS Nano* **2012**, *6* (7), 6400–6406. https://doi.org/10.1021/NN301971R/SUPPL_FILE/NN301971R_SI_001.PDF.

(5) Koga, H.; Tonomura, H.; Nogi, M.; Suganuma, K.; Nishina, Y. Fast, Scalable, and Eco-Friendly Fabrication of an Energy Storage Paper Electrode. *Green Chemistry* **2016**, *18* (4), 1117–1124. https://doi.org/10.1039/C5GC01949D.

(6) Aeby, X.; Poulin, A.; Siqueira, G.; Hausmann, M. K.; Nyström, G. Fully 3D Printed and Disposable Paper Supercapacitors. *Advanced Materials* **2021**, *33* (26), 2101328. https://doi.org/10.1002/ADMA.202101328.

(7) Li, B.; Lopez-Beltran, H.; Siu, C.; Skorenko, K. H.; Zhou, H.; Bernier, W. E.; Whittingham, M. S.; Jones, W. E. Vaper Phase Polymerized PEDOT/Cellulose Paper Composite for Flexible Solid-State Supercapacitor. *ACS Appl Energy Mater* **2020**, *3* (2), 1559–1568. https://doi.org/10.1021/ACSAEM.9B02044/ASSET/IMAGES/LARGE/AE9B02044_0006.JPEG.

(8) Rabani, I.; Park, Y. J.; Lee, J. W.; Tahir, M. S.; Kumar, A.; Seo, Y. S. Ultra-Thin Flexible Paper of BNNT–CNF/ZnO Ternary Nanostructure for Enhanced Solid-State Supercapacitor and Piezoelectric Response. *J Mater Chem A Mater* **2022**, *10* (29), 15580–15594. https://doi.org/10.1039/D2TA03134E.

(9) Soam, A.; Kumar, R.; C, M.; Singh, M.; Thatoi, D.; Dusane, R. O. Development of Paper-Based Flexible Supercapacitor: Bismuth Ferrite/Graphene Nanocomposite as an Active Electrode Material. *J Alloys Compd* **2020**, *813*, 152145. https://doi.org/10.1016/J.JALLCOM.2019.152145.

(10) Chen, I. W. P.; Chou, Y. C.; Wang, P. Y. Integration of Ultrathin MoS2/PANI/CNT Composite Paper in Producing All-Solid-State Flexible Supercapacitors with Exceptional Volumetric Energy Density. *Journal of Physical Chemistry C* **2019**, *123* (29), 17864–17872. https://doi.org/10.1021/ACS.JPCC.9B04046/ASSET/IMAGES/LARGE/JP-2019-04046P_0007.JPEG.
